# Exploring lived experiences of nurses in managing patients with drug-resistant tuberculosis in Western Amhara, Northwest Ethiopia: a descriptive phenomenological study

**DOI:** 10.1186/s12913-025-13887-z

**Published:** 2025-12-23

**Authors:** Abebe Dilie Afenigus, Addise Tariku Kebede

**Affiliations:** 1 Department of Nursing, College of Medicine and Health Sciences, Debre Markos University, PO Box 269, Debre Markos, Gojjam, Ethiopia; 2 Debre Markos Comprehensive Specialized Hospital, Debre Markos, Gojjam, Ethiopia

**Keywords:** Drug-resistant tuberculosis, Nurses lived experience, Descriptive phenomenological study, Western Amhara, Ethiopia

## Abstract

**Background:**

Drug-resistant tuberculosis (DR-TB) remains a major public health challenge due to its complex regimens and high risk of transmission. Nurses play a crucial role in managing DR-TB patients, yet their lived experiences in this demanding context remain underexplored.

**Objectives:**

This descriptive phenomenological study aims to explore the lived experiences of nurses in managing patients with DR- TB in Western Amhara, Northwest Ethiopia.

**Methods:**

A descriptive phenomenological study was conducted across six health institutions in the Western Amhara region. Sixteen nurses directly involved in DR-TB patient care were selected using criterion-based, heterogeneous purposive sampling. Data were collected through an in-depth face-to- face interview. Interviews were audio-recorded, transcribed verbatim, and translated into English, and analyzed using Colaizzi’s seven-step descriptive phenomenological method.

**Results:**

Nurses derived meaning and fulfilment from DR- TB care through patient-centered engagement, collaborative teamwork, ongoing professional development, and providing emotional and practical support. However, they faced significant challenges, including managing patient non-adherence, emotional strain from witnessing patient suffering, and resource constraints that hindered effective care. The psychological impact of DR-TB care was profound, with nurses witnessed patients’ emotional distress and experienced their own emotional fatigue, yet demonstrated resilience through peer support and the satisfaction of seeing patient recovery.

**Conclusion:**

Nurses caring for DR-TB patients navigate a complex interplay of professional fulfillment, systemic challenges, and psychological strain. These findings highlight the critical need for targeted interventions that enhance psychosocial support, expand training opportunities, and address resource gaps. Empowering nurses through multidisciplinary collaboration and continuous professional development is essential to sustain high quality DR-TB care and improve outcomes.

**Supplementary Information:**

The online version contains supplementary material available at 10.1186/s12913-025-13887-z.

## Introduction

Drug-resistant tuberculosis (DR-TB) is a form of tuberculosis that does not respond to first line anti-TB treatments and remains a serious public health threat, continuing to hinder TB control efforts, particularly in high burden countries like Ethiopia [1]. DR-TB categories include isoniazid resistance TB, rifampicin resistant TB(RR-TB), multidrug-resistant TB(MDR-TB), extensively drug-resistant TB(XDR-TB), and pre-XDR TB, which requires bacteriological confirmation of TB and testing for drug resistance using rapid molecular diagnostic tests, culture or sequencing technologies which are limited in Ethiopia [2–5]. Ethiopia remains one of the 30 high TB, TB/HIV, and MDR-TB burden countries, with DR-TB prevalence influenced by socioeconomic factors like poverty, malnutrition, overcrowding and inadequate healthcare infrastructure [6].

In Northwest Ethiopia, adherence to TB treatment remains a challenge, with factors such as low patient knowledge, weak provider and patient relationship, economic and transportation barriers directly affecting treatment outcomes [7]. Non-adherence to TB treatment leads to the emergence and spread of DR-TB, with prevalence rates of non-adherence reaching up to 24.5% in some parts of Ethiopia [8].

Mortality among DR-TB patients treated in Ethiopia ranges from 8% to 30%, with a pooled mortality estimate of 15%, while treatment success rates remain approximately 64% [9]. Despite these sobering statistics, most studies have focused primarily on patient level outcomes and quantitative indicators, while the subjective experiences of nurses delivering DR-TB care in Northwest Ethiopia remain underexplored [10].

The management of DR-TB requires a multidisciplinary approach that integrates clinical expertise with patient-centered care strategies [11]. Nurses play a crucial role in delivered personalized care to DR-TB patients, addressing not only complex medical needs but also the psychosocial and socioeconomic factors influencing treatment outcomes [12, 13]. Their responsibilities include providing counseling, administering treatment regimens, and monitoring patient responses to ensure effective management of the diseases [14, 15]. The complexity of DR-TB treatment such as its high pill burden, potential for severe side effects demands strong nursing response characterized by experience and empathy [14].

However, health care workers (HCWs) in Ethiopian settings often encounter significant challenges in managing DR-TB patients, including fear of infection control measures, human resource constraints and limited training opportunities [10, 16].

Exploring lived experience of nurses in managing DR-TB is essential for enhancing DR-TB care delivery, reducing occupational stress, and improving patient outcomes in resource limited settings such as Northwest Ethiopia. Therefore, the aim of the study was to explore the lived experiences of nurses in managing patients with drug-resistant tuberculosis in Western Amhara, Northwest Ethiopia, with specific reference to meaning, fulfilment and psychological impact of care on nurses and patients alike, and to address clinical and systematic DR-TB management challenges experienced.

## Methods

### Study design

A qualitative approach with descriptive phenomenological study was employed to explore nurses’ lived experiences of best practices and challenges in managing patients with DR-TB in Western Amhara, Northwest Ethiopia. The methodology followed Colaizzi’s seven-step descriptive phenomenological method [17]. Furthermore, the authors strengthen these steps by using bracketing (setting aside personal biases), intuiting (deeply engaging with participants’ experiences), analyzing (identifying themes from meaning units), and describing (articulating the essence of the phenomenon [18–20]. To ensure methodological rigor and transparent reporting, the study adhered to Consolidated Criteria for Reporting Qualitative Research (COREQ) checklist [21].

### Study setting and period

The study is conducted in six healthcare settings across Western Amhara encompassing two comprehensive specialized hospitals, two general hospitals, and two primary hospitals from January to May 2023.

### Study participants and sampling procedure

Participants were nurses in the management of patients with drug-resistant tuberculosis (DR-TB) for at least one year of direct experience in caring for DR-TB patients and expressing consent to participate in the study in the Western Amhara. The researchers used criterion-based, heterogenous purposive sampling to ensure diversity in experience and clinical settings, including comprehensive specialized hospitals, general hospitals, and primary hospitals. A total of 16 participants were included in the study when data saturation, where no new themes or content emerged from subsequent interviews, were reached [22–24].

### Data collection

In-depth interviews were conducted to collect data on the lived experience of nurses’ management of patients with drug-resistant tuberculosis concerning best practices and challenges. All interviews took place in locations mutually agreed upon by the investigators and participating nurses. We collected data through in-depth, face-to-face individual interviews, choosing this method to capture rich and detailed personal experiences in a private setting. This approach allowed us to explore true essence of the phenomenon from each participant’s perspective without bias. We developed an interview guide based on the study’s objectives, covering topics such as sociodemographic information, experience of meaning and fulfillment in managing DR-TB, challenges encountered while caring for DR-TB, patients, and the psychological impact of DR-TB care. The interviewer began the session with open ended questions such as, “can you tell me your experience caring for patients with DR-TB?” to encourage participants to share their experiences in detail. The interviewer followed up with exploratory prompts like “why?”, “how?”, and “can you please explain further?” to clarify and deepen the discussion. The interviews were conducted in Amharic, the local language, to support open and meaningful discussions. Each interview session lasted approximately 60–80 min and was audio recorded with the participant’s consent. At the end of each interview, the interviewer informed that a follow-up phone call might be necessary to discuss the study findings and to confirm that the findings accurately reflect the participant’s experience. The first author, who had prior training and experience in qualitative research methods, conducted all interviews. To minimize bias and potential power dynamics, the investigators made sure they were not direct colleagues of participants and did not hold any supervisory roles over them. Due to limited number of interviewers and constraints related to logistics and budget, we conducted the interviews sequentially across six different health care settings.

### Data analysis

Data analysis followed Colaizzi’s seven-step descriptive phenomenological method, a well-established approach in qualitative research [17]. All interviews were audio-recorded, then the audio-recorded interviews were transcribed from Amharic and translated into English by investigators to prepare the data. The analysis process involved: [1] bracketing to set aside researchers’ personal biases [2], repeatedly reading transcripts to gain deep familiarity with participants experience [3], extracting significant statements relevant to the phenomenon [4], formulating meanings from these statements [5], clustering meanings in to themes [6], developing a comprehensive description of the phenomenon, and [7], identifying and verifying the fundamental structure [17], (Table 2). In this study, clustering themes refer to the grouped meaning units derived from Colaizzi’s method, used interchangeably with ‘themes’ to describe nurses’ lived experiences. To minimize bias and strengthen credibility, an independent qualitative expert reviewed a subset of transcripts and collaborated with the principal investigators in theme development through discussion and consensus. Any differences in interpretation were resolved through dialogue to ensure the trustworthiness of the findings.

### Trustworthiness

To ensure trustworthiness of the findings, multiple strategies were implemented. Credibility was achieved through prolonged interaction with participants, allowing investigators to build rapport and gain an in-depth understanding of their experiences. Reflexivity was also practiced consistently, with investigators actively identifying and managing their own biases throughout the study [25]. Transferability was supported by providing rich, detailed descriptions (tick description) of the research context, participants and procedures to allow readers to assess applicability to other settings [25]. Dependability was maintained by keeping a thorough audit trail documenting all research processes, decisions, and analytical steps [25]. Finally, confirmability was achieved through peer debriefing with an independent coder who reviewed and validated the findings, alongside member checking, where participants reviewed & confirmed their statements, helping to reduce researcher bias [25].

## Results

### Sociodemographic characteristics

Sixteen nurses participated in this study on the nursing management of patients with drug-resistant tuberculosis (DR-TB). The participant’s ages ranged from 30 to 50 years and the mean age of the participants was 36 years with a standard deviation (SD) of 1.5. In terms of DR-TB care experience, 12 participants had 3–5 years of experience. Besides this, 10 participants were from specialized hospitals (Table 1).

**Table 1:** Sociodemographic characteristics of the respondents

Characteristics	Category	Frequency
Gender	Male Female	12 4
Age group Mean = 36; SD = 1.5	30–40 years 40–50 years	10 6
Marital status	Single Married	4 12
Experience in DR- TB nursing care	1–2 years 3–5 years	4 12
Nurses employed in healthcare settings	Comprehensive specialized hospital General hospital Primary hospital	10 4 2

### Clustered themes identified

Using Colaizzi’s phenomenological method, nurses’ lived experiences in managing DR-TB patients were grouped into three major clustered themes: [1] experiencing meaning and fulfillment in DR-TB nursing care [2], struggling within constraints: lived challenges of nurses, and [3] living the psychological impact of DR-TB care. Each clustered theme encompasses specific sub-themes, supported by participant quotes (Table 2).

**Table 2:** Clustered themes and subthemes of nurses lived experiences in managing patients with DR-TB in Western Amhara, Ethiopia

No.	Clustered theme	Sub-themes	Supporting quotes
**1**	Experiencing meaning and fulfillment in DR-TB nursing care	Experiencing trust-building through patient-centered engagement	“Patient-centered care for DR-TB involves understanding each patient’s unique circumstances… building trust and rapport…” (P1) “We involve patients in decision-making regarding their treatment plan…” (P6)
		Feeling empowered by collaborative teamwork	“Our healthcare team adopts a multidisciplinary approach… regular team meetings…” (P2) “We had a patient with extensively drug-resistant TB… collaborated closely…” (P9)
		Growing professionally through learning opportunities	“I participated in refreshment seminars… focusing on the latest treatment guidelines…” (P1) “Training has equipped me with the knowledge to confidently manage DR-TB patients…” (P12)
		Finding purpose in providing emotional and practical support	“We establish open communication with patients and their families…” (P5) “Support groups are instrumental in providing peer support…” (P9)
**2**	Struggling within constraints: Lived challenges of nurses	Carrying the emotional burden of patient non-adherence	“Managing treatment adherence… is a daily challenge…” (P1) “Stigma associated with tuberculosis is a significant barrier…” (P15)
		Balancing clinical responsibilities with patient suffering	“Managing side effects is a constant challenge…” (P3) “We collaborated with a nutritionist to develop a diet plan…” (P3)
		Coping with resource scarcity and systemic limitations	“Limited resources impact every aspect of our care…” (P1) “Inadequate staffing levels and heavy workload…” (P5)
**3**	Living the psychological impact of DR-TB care	Witnessing the psychological burden on patients	“DR-TB patients often experience fear and anxiety due to stigma…” (P13) “Many patients experience feelings of depression…” (P7)
		Experiencing emotional fatigue and resilience as nurses	“Managing DR-TB patients can be emotionally draining…” (P5) “Despite the challenges, there’s a sense of fulfillment…” (P6)

### Clustered theme 1: experiencing meaning and fulfillment in DR-TB nursing care

In managing patients with drug-resistant tuberculosis (DR-TB), nurses experiencing meaning and fulfilment in DR-TB nursing care, which has four sub-themes: Experiencing trust-building through patient-centered engagement, feeling empowered by collaborative teamwork, growing professionally through learning opportunities, and Finding purpose in providing emotional and practical support.

### Sub- theme 1: experiencing trust-building through patient-centered engagement

Participants consistently emphasized the importance of patient-centered care in the management of DR-TB, highlighting strategies such as building trust, involving patients in decision-making, and providing care based on individual needs. Nurses experiencing a deeper connection with patients when they took time to understand and respect their unique circumstances and preferences, which they saw as central to effective care delivery.

*Patient-centered care for DR-TB involves understanding each patient’s unique circumstances and preferences. We start by building trust and rapport*,* ensuring patients feel heard and respected.* This initial step of relationship-building was seen as foundational in promoting patient engagement and adherence (P1)

Nurses also noted that involving patients in their own care decisions is both empowering and improving treatment outcomes.

*We involve patients in decision-making regarding their treatment plan. This includes discussing treatment options*,* addressing concerns about side effects*,* and collaborating to develop a plan that aligns with their lifestyle and goals. (P6)*

Listening attentively and adapting care based on patient feedback were also a key component.

*Active listening is key. We encourage patients to share their concerns and preferences openly. This helps us modify our approach and adapt the care plan to meet their individual needs. (P10)*

Education and communication on patients’ condition were vital.

*Educating patients about their condition and treatment options empowers them to make informed decisions. We use visual aids and simplified explanations to enhance understanding and promote adherence. (P4)*

Understanding the broader context of each patient’s life was viewed as essential for delivering meaningful and effective care.

*Each patient is unique*,* so we take the time to understand their background*,* beliefs*,* and challenges. This helps us create a care plan that they are comfortable with and motivated to follow. (P8)*

In addition to initial engagement, participants stressed the importance of continuous improvement through feedback and adaptation.

*Regularly assessing patient satisfaction and adjusting our approach based on their feedback is crucial. It shows patients that their input is valued and helps us continuously improve our care delivery. (P6)*

### Sub-theme 2: feeling empowered by collaborative teamwork

Nurses emphasized feeling empowered and supported when working closely with professionals from different disciplines to provide comprehensive and coordinated care.

*Our healthcare team adopts a multidisciplinary approach where each member contributes their expertise. We have regular team meetings to discuss patient progress*,* treatment adjustments*,* and any challenges encountered.(P2).* These routine discussions and joint decision-making processes were highlighted as a key for ensuring timely and patient-specific interventions

Consistent communication in patient handover was an important process in maintaining care continuity.

*Communication is key. We use patient charts and patient handovers to ensure all team members are updated on patient status and care plans. This helps streamline care delivery and ensures continuity. (P3)*

Participants outlined the distinct contributions of different team members and how their roles intersect to form a unified care framework.

*Doctors oversee the medical management*,* including prescribing and monitoring medications. Pharmacists play a crucial role in ensuring the availability of medications and educating patients about their treatment. (P5)*

Beyond the clinical staff, nurses acknowledged the value of community care and psychosocial support.

: *Health extension workers provide invaluable support by addressing social determinants of health*,* such as housing stability and access to financial resources. They also offer counseling and facilitate patient education sessions on adherence and disease management. (P11)*

In describing the practical impact of collaboration, nurses shared specific cases that highlighted the power of team work in achieving successful outcomes.

*We had a patient with extensively drug-resistant TB who required complex treatment regimens and close monitoring. Our team*,* including physicians*,* nurses*,* pharmacists*,* laboratory workers*,* and others*,* collaborated closely to give a comprehensive care plan. This included regular check-ins*,* adjustments in treatment based on microbiological results*,* and continuous support to manage side effects and psychosocial challenges.(P9)*

Similarly, coordinated efforts were instrumental in community-level interventions and infection control.

* Our multidisciplinary team worked together to implement infection control measures*,* conduct contact tracing*,* and ensure prompt diagnosis and treatment initiation for affected individuals. This coordinated effort helped contain the spread of DR-TB and provided timely support to affected patients. (P3)*

### Sub-theme 3: growing professionally through learning opportunities

Continuous professional development by taking training was identified by participants as a vital component in equipping nurses with the necessary knowledge and skills to manage the complexities of drug-resistant tuberculosis (DR-TB). Nurses described various learning opportunities that helped them stay updated on evolving treatment guidelines and enhance their clinical practice.

*I participated in refreshment seminars organized by our healthcare facility*,* focusing on the latest treatment guidelines and infection control measures specific to DR-TB. These sessions provided updates on emerging therapies and practical strategies for patient care.(P1)*

Similarly, nurses also highlighted formal in-service training opportunities as key contributors to professional development.

*As part of my ongoing professional development*,* I took training on tuberculosis management offered by in-service training centers. This training covered topics such as drug resistance mechanisms*,* treatment protocols*,* and patient counseling. (P4)*

Nurses reflected on how these experiences shaped their growth, confidence, and ability to manage complex patient needs.

*Training has equipped me with the knowledge to confidently manage DR-TB patients*,* from understanding the complexities of treatment regimens to recognizing and managing potential complications. It has improved my ability to provide comprehensive care and support to patients. (P12)*

Moreover, education(training) emphasized not only clinical skills but also the importance of collaborative and patient-centered approaches.

*The training emphasized the importance of multidisciplinary collaboration and patient-centered care. It has encouraged me to involve patients in treatment decisions and to advocate for their holistic needs beyond medical management. (P1)*

Despite these benefits, nurses also identified gaps where further training could enhance their effectiveness in DR-TB care.

*Advanced training on anti-TB medications would be beneficial. Understanding the mechanisms of action of different drugs and resistance patterns would enable us to optimize treatment plans and manage side effects more effectively. (P3)*

Additionally, there was a recognized need for strengthening psychosocial support competencies.

*More resources for psychosocial support and counseling training would be invaluable. Many DR-TB patients face stigma and psychological challenges that require sensitive and skilled interventions from healthcare providers. (P10)*

### Sub-theme 4: finding purpose in providing emotional and practical support

Nurses emphasized the critical role of supportive care and counseling in addressing both the medical and emotional needs of patients with DR-TB and their families. From the outset, they prioritize establishing open communication, ensuring patients and their families are well-informed and emotionally supported throughout the treatment journey.

*We establish open communication with patients and their families from the beginning. This involves regular counseling sessions where we discuss the treatment plan*,* potential side effects*,* and coping strategies. (P5)*

*Beyond individual counseling*,* nurses highlighted the importance of peer support networks in reducing isolation and building a sense of community among patients and their families.*

*Support groups are instrumental in providing peer support and reducing feelings of isolation. We facilitate group sessions where patients and families can share experiences and encourage each other. (P9)*

Nurses experienced patients’ concerns about the lengthy treatment process as a shared emotional challenge, which they addressed through proactive communication.

*Patients often worry about the duration of treatment and the impact on their daily lives. We provide detailed explanations about the treatment timeline and offer strategies to integrate treatment into their routines. (P13)*

Families, meanwhile, frequently seek reassurance about transmission risks and safety precautions.

*Families often express concerns about transmission and safety. We educate them about infection control measures and emphasize the importance of adherence to treatment to minimize risks.(P2)*

Nurses also shared patient stories illustrating how sustained counseling positively influenced patients’ acceptance of their diagnosis and commitment to treatment.

*I recall a young patient who initially struggled with accepting his diagnosis and adhering to treatment. Through consistent counseling and encouragement*,* we helped him understand the importance of treatment and supported his emotional journey. Eventually*,* he became more engaged in his care and showed remarkable improvement. (P3)*

Supporting families emotionally and practically to strengthen their coping abilities was also important.

*Supporting families is also essential. I remember a case where we worked closely with a patient’s family to address their fears and provide practical support. By involving them in the care plan and offering emotional support*,* we strengthened their ability to cope with the challenges of DR-TB. (P6)*

### Clustered theme 2: struggling within constraints: lived challenges of nurses

In managing patients with drug-resistant tuberculosis (DR-TB), nurses struggled with constraints, which has three sub-themes: carrying the emotional burden of patient non-adherence, balancing clinical responsibilities with patient suffering, and coping with resource scarcity and systemic limitations.

### Sub-theme 1: carrying the emotional burden of patient non-adherence

Participants shared diverse experiences that highlight the complexity of sustaining adherence to the long and demanding treatment regimens required for DR-TB. One nurse reflected on the daily struggle:

*Managing treatment adherence among DR-TB patients is a daily challenge. Many patients struggle with the sheer number of pills they have to take each day and the duration of treatment. It’s not uncommon for patients to miss doses or discontinue treatment prematurely. (P1)*

The variation in patient commitment was also noted:

*Our experience varies depending on the patient’s understanding of their condition and commitment to treatment. We see patients who are diligent with their medications*,* but others face significant barriers that affect adherence. (P8****)***

Nurses described the emotional toll of navigating the multiple barriers that hinder patient adherence.

*One of the biggest barriers is medication side effects. Many patients experience nausea*,* dizziness*,* and hearing problems*,* which can be severe enough to make them reluctant to continue treatment. (P3)*

Socioeconomic challenges further exacerbate adherence difficulties:

*Socioeconomic factors also play a major role. Patients may struggle to afford transportation costs to clinics for regular check-ups or may have competing priorities that make it difficult for them to prioritize their health. (P4)*

Stigma associated with tuberculosis was another critical barrier impacting patients’ willingness to adhere:

*Stigma associated with tuberculosis is another significant barrier. Patients often feel ashamed and may hide their diagnosis from family and friends*,* impacting their willingness to adhere to treatment. (P15)*

Moreover, continuous follow-up and building trust were seen as vital:

*Regular follow-up appointments and phone reminders are essential. We try to build strong relationships with our patients to create a supportive environment where they feel comfortable discussing any challenges they face with their treatment. (P7)*

Providing support, especially for patients facing financial difficulties due to comorbidities, was highlighted as essential in overcoming treatment barriers.

*Although anti-TB medications are provided free of charge*,* patients with comorbidities and financial constraints often face additional medication expenses. In such cases*,* we connect them with social workers to help secure waivers for these costs. Addressing these concerns and individual priorities is crucial for improving adherence. (P1)*

### Sub-theme 2: balancing clinical responsibilities with patient suffering

Nurses reported that managing the side effects of DR-TB medications is one of the most persistent and challenging aspects of care. Given the intensity and duration of DR-TB treatment regimens, participants emphasized the importance of vigilant monitoring and proactive intervention to ensure patient comfort and continued adherence.

*Managing side effects is a constant challenge. We closely monitor patients for any signs of adverse reactions and adjust medications accordingly. For instance*,* if a patient experiences severe nausea*,* we might recommend taking the medication with food or splitting the dose throughout the day. (P3)*

Effective report was also highlighted as key to early detection and timely management of adverse effects.

*We encourage patients to report any side effects they experience promptly. This helps us intervene early to minimize discomfort and ensure they can continue their treatment regimen effectively. (P8)*

In addition to managing side effects clinically, nurses described feeling more effective and less overwhelmed when they could prepare patients through targeted education, which plays a vital role in reducing fear and increasing treatment adherence.

*During initial consultations*,* we discuss the common side effects of DR-TB medications in detail. We provide information about what to expect*,* such as reduced hearing or gastrointestinal issues*,* and discuss strategies to minimize their impact. (P11)*

Visual tools and printed materials were used to support learning and retention, especially in settings where health literacy might be limited.

*We use visual aids and pamphlets to reinforce key information about side effects. We encourage patients to ask questions and clarify any concerns they may have*,* ensuring they feel confident in managing their treatment. (P4)*

Participants shared specific experiences that illustrated the need for flexibility, interdisciplinary collaboration, and continued support in managing treatment-related complications.

*I recall a patient who experienced severe dizziness and headaches shortly after starting treatment. We worked closely with the medical team to adjust the dosage and provided supportive care to alleviate the symptoms. Through consistent monitoring and adjustments*,* we were able to help the patient tolerate the medication. (P12)*

Another participant emphasized the value of involving nutritionists in care to address side effects that affect patients’ overall health.

*One patient struggled with persistent nausea*,* which affected their appetite and overall well-being. We collaborated with a nutritionist to develop a diet plan that helped alleviate nausea and ensure the patient received adequate nutrition to support their recovery. (P3)*

### Sub-theme 3: coping with resource scarcity and systemic limitations

Nurses consistently reported facing challenges due to inadequate resources. These included shortages of essential medications for DR-TB treatment, diagnostic tools such as GeneXpert machines for rapid TB testing, and personal protective equipment (PPE) necessary for infection control. The scarcity of these resources often compromised the quality of care provided to DR-TB patients and posed significant barriers to effective management strategies. Besides limited material resources, participants also highlighted the burden of high patient caseloads coupled with a shortage of nursing staff. This resulted in nurses having to manage multiple patients simultaneously, leading to difficulties in providing individualized care and spending adequate time with each patient.

*Limited resources impact every aspect of our care. We often face shortages of essential medications and diagnostic tools needed for timely treatment initiation and monitoring. This delays patient care and can affect treatment outcomes. (P1)*

*Infrastructure constraints in our facility mean we don’t always have enough isolation rooms or proper ventilation for DR-TB patients. This compromises infection control measures and poses risks to both patients and healthcare workers. (P10)*

Moreover, participants discussed the direct impact of resource constraints on patient outcomes and their daily practice:

*Delayed diagnosis due to limited access to rapid diagnostic tests means patients may not receive appropriate treatment early enough. This can lead to disease progression and poorer prognosis. (P3)*

*Inadequate staffing levels and heavy workload due to resource shortages affect our ability to provide individualized care. We often have to prioritize tasks*,* which can result in compromised quality of care. (P5)*

Nurses shared how they adapted and coped with the stress of inadequate resources, drawing on collaboration and advocacy.

*We’ve established drug supply chain management with zonal health departments and regional health bureau to secure emergency supplies of medications during shortages. This ensures continuity of treatment for our patients. (P11)*

*To address infrastructure challenges*,* we’ve advocated for improved facility planning and allocation of funds for renovations. We’ve also implemented strict infection control protocols to minimize the risk of transmission in crowded settings. (P16)*

### Clustered theme 3: living psychological impact of DR-TB care

In managing patients with drug-resistant tuberculosis (DR-TB), nurses identified psychological impacts on patients and themselves, which has two sub-themes: witnessing the psychological burden on patients and experiencing emotional fatigue and resilience as nurses.

### Sub-theme 1: witnessing the psychological burden on patients

Nurses often witnessed and internalized their patients’ fear, anxiety, and isolation caused by stigma, which deeply affected how they related to their patients.

*DR-TB patients often experience fear and anxiety due to the stigma associated with tuberculosis. They may feel isolated and misunderstood by their community*,* which adds to their psychological burden. (P13)*

The prolonged duration of treatment, coupled with concerns about potential side effects, contributes to patients’ feelings of uncertainty and despair.

*Patients also struggle with uncertainty about their future health outcomes. The long duration of treatment and potential for side effects can lead to feelings of hopelessness and despair. (P2)*

Emotional distress among patients manifests as depression and low self-esteem, often in response to the difficult diagnosis and demanding treatment regimen.

*Many patients experience feelings of depression and low self-esteem as they hear the diagnosis and treatment regimen. We provide emotional support through counseling sessions and encourage peer support groups where patients can share their experiences. (P7)*

Empathy and interpersonal communication skills were described as key tools in helping patients manage these challenges and remain committed to their treatment.

*Empathy and active listening are crucial in supporting patients emotionally. We validate their concerns and provide reassurance*,* emphasizing the importance of adherence to treatment and the potential for recovery. (P4)*

#### Sub-theme 2: experiencing emotional fatigue and resilience as nurses

Nurses described feeling emotionally drained, yet also found strength and meaning through team support and patient recovery.

*Managing DR-TB patients can be emotionally draining. Witnessing their struggles with the disease and the impact it has on their lives is difficult. It’s important for us as a team to support each other and debrief regularly to process our emotions. (P5)*

Despite these challenges, nurses found strength in team cohesion, mutual support, and celebrating patient progress, which fostered personal and professional fulfillment.

*Despite the challenges*,* there’s also a sense of fulfillment in helping patients navigate their journey towards recovery. We draw strength from each other and celebrate small victories along the way. (P6)*

## Discussion

This study explored the lived experiences of nurses providing care for DR‑TB patients in Western Amhara, Ethiopia. Nurses managing patients with DR-TB experienced a profound sense of meaning and fulfillment through building trust with patients, engaging in collaborative teamwork, professional growth opportunities, and providing emotional and practical support. However, they also faced significant challenges, including the emotional burden of patient non-adherence, balancing demanding clinical responsibilities with patient suffering, and coping with resource shortages and systemic limitations. Psychologically, nurses witnessed the heavy mental burden on patients caused by stigma and uncertainty, while also grappling with their own emotional fatigue. Despite these difficulties, nurses demonstrated resilience, drawing strength from team support and patient progress, highlighting the complex but rewarding nature of DR-TB nursing care.

Nurses emphasized the importance of empathy, individualized communication, and trust to patients to foster adherence and meaningful care. A multi- national study from Georgia, Mongolia, and South Africa also identified good relationships with clinical staff as essential for alleviating treatment-related fears, reinforcing the role of relational care in multiple settings [26]. Our participants derived strength from multidisciplinary teamwork, involving pharmacists, doctors, and community workers. This mirrors a qualitative study in South Africa, where healthcare providers described the decentralized implementation of DR‑TB care as “being dropped on us with minimal system coordination” which highlights the critical need for structured integration and support [27].

Moreover, nurses found personal fulfillment in counseling and peer support facilitation for DR‑TB patients and their families. Similar observations have been made in Nigeria, where hospitalized MDR‑TB patients often rely more on peer emotional support than strained provider relationships, suggesting the potential value of structured peer-facilitated care models [28].

Emotionally strain was evident as nurses faced the challenges of patients’ non-adherence, often driven by medication side effects, socioeconomic hardship, and stigma. Multinational research from South Africa, Georgia, and Mongolia similarly documented intense patient distress including fear, stigma, and uncertainty which complicates adherence and caregiver burden [26]. Managing severe side effects and providing ongoing education stretched nursing capacities. Globally, DR‑TB patients report high pill burdens, and debilitating side effects, mirroring the dual clinical and emotional strain experienced by nurses [29].

Systemic challenges such as shortages in diagnostics, infection control infrastructure, and staffing were also reported. These limitations are common in sub-Saharan Africa; for example in Uganda, the limited number of DR‑TB initiation sites led to treatment waiting lists prior to program expansion [30], while in Nigeria, overcrowded wards and infrastructure deficits contributed to heightened psychological trauma [28].

Nurses often witnessed patients’ emotional distress, including despair and isolation. Consistent with global studies, DR‑TB patients often experience depression, suicidal ideation, stigma, and fear of transmitting the diseases, particularly in settings like Kolkata and Bangladesh [29, 31]. Despite the emotional exhaustion reported, nurses also experienced moments of fulfillment when patients improved. A broad narrative review in Africa confirmed that TB care providers face chronic stress but derive resilience and strength from patient recovery and team support [15].

### Implications for practice and policy

The findings highlight critical implications for both policy and practice in DR-TB care. Policymakers should prioritize strengthening multidisciplinary collaboration by formalizing integrated care models that include not only clinical staff but also community health workers (health extension workers) to ensure holistic patient management. Investment in continuous professional development is essential to equip health care providers with up-to-date knowledge and skills. Addressing systemic resource limitations such as shortages in diagnostics, infrastructure, and staffing is imperative to enhance care quality, reduce treatment delays, and improve infection control. Furthermore, policies should support the development of structured peer-support programs to alleviate patient isolation and stigma, which are major barriers to adherence. At the practice level, fostering patient-centered communication and trust-building is crucial to empower patients and improve treatment engagement. Lastly, recognizing and addressing the psychological impact on both patients and providers(nurses) through regular emotional support and team debriefings can mitigate burnout and promote resilience, ultimately enhancing the sustainability and effectiveness of DR-TB care delivery. Future studies could explore artificial intelligence (AI) driven interventions for DR-TB management, such as machine learning for treatment adherence monitoring, and investigate cross-country comparisons of nurse-led DR-TB care.

### Strength and limitation of the study

### Strength of the study

This study used strong qualitative methodology, using descriptive phenomenological design and criterion-based, purposive sampling, allowing for in-depth exploration of lived experiences of nurses in managing DR-TB. The use of Colaizzi’s seven-step ensured a systematic and rigorous data analysis. Bracketing and reflexivity were applied throughout the research to minimize researcher induced bias and enhance credibility. The study adheres to COREQ checklist, further supporting methodological transparency and rigor.

### Limitations of the study

The study has a limited sample size and includes participants from a restricted geographic and demographic scope, which may affect the transferability of our findings. Finally, the study relied on self-reported experiences, which could be influenced by social desirability bias, though efforts were made to foster openness and trust during interviews.

## Conclusion

In conclusion, nurses managing DR-TB patients experience a deep sense of fulfillment through trust-building, teamwork, professional growth, and emotional support, despite facing significant challenges such as patient non-adherence, resource constraints, and emotional fatigue. Their resilience and commitment emphasize the importance of empathetic, patient-centered care and multidisciplinary collaboration. Addressing systemic barriers and providing ongoing psychological and professional support are essential to improve both nurse well-being and patient outcomes in DR-TB care.

## Supplementary Information

Below is the link to the electronic supplementary material.

Supplementary Material 1

## Data Availability

The data that support the findings of this study are available from the corresponding author upon reasonable request.

## Abbreviations

COREQ: Consolidated Criteria for Reporting Qualitative Research
DR-TB: Drug Resistance Tuberculosis
MDR-TB: Multi-Drug Resistance Tuberculosis
XDR-TB: Extensively Drug-Resistant Tuberculosis
pre-XDR TB: Pre-Extensively Drug-Resistant Tuberculosis
