# Supplementary material for: Exploring lived experiences of nurses in managing patients with drug-resistant tuberculosis in Western Amhara, Northwest Ethiopia: a descriptive phenomenological study
Source: BMC Health Serv Res. 2025 Dec 23;26:121. doi: 10.1186/s12913-025-13887-z (PMC12837119; doi:10.1186/s12913-025-13887-z)
Supplement: Supplementary file 1 — Supplementary Material 1 [file 12913_2025_13887_MOESM1_ESM.docx]

**Interview Guide for DR-TB**

**Title:** Exploring lived experiences of nurses in managing patients with drug-resistant tuberculosis in Western Amhara, Northwest Ethiopia: A descriptive phenomenological study

**Objective:** This descriptive phenomenological study aims to explore the lived experiences of nurses in managing patients with DR- TB in Western Amhara, Northwest Ethiopia

**Introduction:**

- Welcome and thank the participant for their time.
- Briefly introduce yourself and explain the purpose of the interview: to explore the lived experiences of nurses in managing patients with DR- TB in Western Amhara, Northwest Ethiopia.
- Ensure confidentiality and obtain informed consent.

**Sociodemographic information**

- Age, gender, marital status, years of experience in nursing.
- Current healthcare setting

Main interview

- Can you describe what gives you meaning and fulfillment in your role managing patients with DR-TB?
- Can you describe your experience with managing DR-TB patients?
- What are the common barriers you encounter in caring DR-TB patients?
- How do you currently address or mitigate these challenges?
- How do you manage the side effects of DR-TB medications?
- What strategies do you use to educate patients about potential side effects and their management?
- Can you share any specific experiences or examples related to side effects management?
- In what ways do limited resources impact your ability to provide care for DR-TB patients?
- How do these limitations affect patient outcomes or your daily practice?
- Have you developed any strategies to cope with resource constraints?
- How do you perceive the psychological impact of DR-TB on patients?
- What emotional challenges do patients typically face, and how do you support them?
- How does managing DR-TB patients affect you and your colleagues emotionally?
- Can you describe how you incorporate patient-centered care in managing DR-TB patients?
- What specific strategies or approaches do you find most effective in providing patient-centered care?
- How does your healthcare team collaborate in managing DR-TB patients?
- What roles do different team members (e.g., doctors, pharmacists, social workers) play in the care of DR-TB patients?
- Can you share an example of a successful multidisciplinary approach in DR-TB management?
- What training or educational opportunities have you received related to DR-TB management?
- How has this training influenced your practice?
- What additional training or resources do you think would benefit nurses in managing DR-TB?
- How do you provide supportive care and counseling to DR-TB patients and their families?
- What are some common concerns or questions raised by patients and families, and how do you address them?
- Can you share a memorable experience where supportive care made a significant difference for a patient or family member?

**4. Closing:**

- Thank the participant for their insights and participation.
- Offer an opportunity for the participant to add any additional comments or insights they feel are important.
- Confirm their willingness to be contacted for follow-up questions if needed.
